# A Review on Therapeutic Drug Monitoring of Immunosuppressant Drugs

**Published:** 2011

**Authors:** Niloufar Mohammadpour, Sepideh Elyasi, Naser Vahdati, Amir Hooshang Mohammadpour, Jamal Shamsara

**Affiliations:** 1 *Department of Microbiology, School of Medicine, Islamic Azad University, Mashhad Branch, Mashhad, Iran*; 2 *Zakariya Research Centre, Islamic Azad University, Mashhad Branch, Mashhad, Iran*; 3 *Department of Pharmacodinamy and Toxicology, School of Pharmacy, Mashhad University of Medical Sciences,Mashhad, Iran*; 4 *Pharmaceutical Research Centre, Mashhad University of Medical Sciences, Mashhad, Iran*; 5 *Department of Biotechnology, School of Pharmacy, Mashhad University of Medical Sciences*,* Mashhad, Iran*

**Keywords:** Cyclosporine, Mycophenolic acid, Sirolimus, Tacrolimus, Therapeutic drug monitoring

## Abstract

Immunosuppressants require therapeutic drug monitoring because of their narrow therapeutic index and significant inter-individual variability in blood concentrations. This variability can be because of factors like drug-nutrient interactions, drug-disease interactions, renal-insufficiency, inflammation and infection, gender, age, polymorphism and liver mass. Drug monitoring is widely practiced especially for cyclosporine, tacrolimus, sirolimus and mycophenolic acid.

**Cyclosporine:**

Therapeutic monitoring of immunosuppressive therapy with cyclosporine is a critical requirement because of intra- and inter-patient variability of drug absorption, narrow therapeutic window and drug induced nephrotoxicity.

**Mycophenolic acid (MPA):**

Some reasons for therapeutic drug monitoring of MPA during post-transplant period include: relationship between MPA pharmacokinetic parameters and clinical outcomes, Inter-patient pharmacokinetic variability for MPA despite fixed MMF doses, alternations of MPA pharmacokinetics during the first months after transplantation, drug- drug interaction and influence of kidney function on MPA pharmacokinetic.

**Sirolimus:**

A recent review of the pharmacokinetics of sirolimus suggested a therapeutic range of 5 to 10 μg l^−1^ in whole blood. However, the only consensus guidelines published on the therapeutic monitoring of sirolimus concluded that there was not enough information available about the clinical use of the drug to make recommendations.

**Tacrolimus:**

Sudies have shown, in kidney and liver transplant patients, significant associations of low tacrolimus concentrations with rejection and of high concentrations with nephrotoxicity. Although the feasibility of a limited sampling scheme to predict AUC has been demonstrated, as yet, trough, or pre-dose, whole blood concentration monitoring is still the method of choice.

## Introduction

In order to reach the optimum balance between therapeutic efficacy and the occurrence of adverse effects, a physician has to individualize a patient’s drug therapy. As all the patients vary in both pharmacokinetics (PK) and pharmacodynamic (PD), obtaining to the goal is always very complicated (1). Thus, the lack of control of drug concentration prevents the patient’s clinical response variability when both PK and PD variability are considerable (2-5). In the early 1960s the measurement of the low drug concentrations found in biological fluids during drug treatment was available by using new analytical techniques. Therefore, the process which was known as therapeutic drug monitoring, provided the chance to reduce the pharmacokinetic component of variability by controlling drug therapy using concentrations in the body rather than just by dose (TDM) (1,6). 

A suitable drug should be chose for therapeutic drug monitoring based on the following criteria: 

1) The relationship between drug concentration and the effect of the drug should be cleared. 

2) Narrow therapeutic index; which means the concentrations of separation the drug make therapeutic benefit and those causing adverse effect should be small.

3) The difference between-subject pharmacokinetic variability and a poor relationship between dose and drug concentration/response should be noticeable. 

4) The assessment of the pharmacological response of the drug from the adverse events should be difficult to obtain (7).

The aim of a therapeutic window, especially in combination regimens, should be defined by avoiding under exposure with an increased risk of rejection or ever exposure with an increased risk of toxicity. First, as the risk of rejection diminishes over time, the therapeutic window for an immuno suppressive drug may also vary overtime. 

Furthermore, as demonstrated for CNI-induced nephrotoxicity, the cumulative risk for toxicity may increase overtime (8,9).

Most maintenance patients (beyond the first year) can be treated in 2 ways: 1) drug levels derived from a therapeutic window, which can be obtained from the study after transplantation, 2) drug levels derived from an opinion-based therapeutic window. 

Second, based on the synergistic of the immunosuppressants for rejection prophylaxis and the potential of their toxicities, the therapeutic window for a given drug in a multi-drug regimen may vary (10).

Additional factors, which can affect the efficacy and toxicity of an immunosuppressive regimen, are donor and recipient characteristics (such as age, number of mismatches, race, delayed graft function, real-and liver function).

Next, the logic of TDM is based on the theory that a proportional increase in dose would results in a proportional increase in exposure.

Although data in maintenance populations may show such linearity, in some cases such as early post-transplant period, the absorption of drugs may change obviously and may not be linear. 

Examples are the absorption pattern for CNIS and mycophenolic Acid (MPA), which undergo considerable changes in the early transplant period (11,12). For example in the first weeks after transplantation, a doubling of MPA dose would result only in approximately 50% in MPA exposure (13,14).

PD assessment can be achieved by biomarkers which obtained of the investigation of the mechanism of action of immunosuppressive drugs. These biomarkers act specifically for a drug like the target enzyme, for example IMPDH for MPA (19). Calcineurin activity is the other drug target which was studies in transplant patients who received calcineurin inhibitors, i.e., cyclosporine A (CyA) (20,21).

Cytokines were also studied as PD biomarkers by different research groups.

Rostaing *et al* investigated the PD influences on cytokines in PBMCs by flow cytometry and found differences in the TH1/TH2 cytokine expression pattern from CyA or tacrolimus treated stable renal allograft recipients compared with healthy volunteers (22,23). In the other word, cytokine’s activated gene expression can be used as a pharmacodynamic readout of immunosuppression (24). 

The measurement of the expression and down-regulation of cell surface markers as an estimate of cell cultivation and T-cell function via cytokine expression (25-27) or overall ATP content also can be used (6,28).

The purpose of this review is to examine the current strategies in use for the therapeutic drug monitoring of immunosuppressant drugs and to discuss some of the factors that impinge on the monitoring of these drugs simply and briefly for clinicians and pharmacists.

***Cyclosporine A***

In, 1969, the early days of kidney transplantation, there was less than 18% possibility for the 2 years graft survival rate for cadaveric renal transplantation in the USA (29). Five years later, in 1974, Caln (the same author of the previous report) reported that the graft survival for 2 years had developed to over 50% (30). This change in the survival rate was because of better surgical technique and improvement in patient management, but the main drug therapies, azathioprine and prednisolone maintained the same. During the past 16 years, the pharmacological change of the immune system in the field of solid-organ transplantation has undergone remarkable improvements. For example, 1 year kidney allograft survival now approaches 95% for transplanted living related kidneys and 85-90% for cadaveric kidneys (31), whereas before the introduction of CsA. 1 year graft survival was 60%. 

Organized investigation on the PK and TDM of immunosuppressive agents began early in the “CsA era” of immunosuppressive therapy in 1683 (6, 32-37). Different absorption and narrow therapeutic index (the drugs which cause irreversible kidney damage when given in extra dose) have lead to the assessment of CsA blood concentrations in order to manage the drug dosage (38). 

Two target ranges have been used by most centers. The first one for preliminary therapy (usually up to 6 months posttransplant), and the second for maintenance therapy using lower, target range treatment thereafter. The target ranges differ in the analysis method, transplant type, and transplant center philosophy on approvable immunosuppression intensity. The retrospective review of CsA concentration data and its correlation with clinical events from single-center studies can improve target ranges (39). 

In the past, the choice of sample matrix for monitoring (40,41), lack of assay specificity (42) inconsistent assay performance (43) the variable absorption of the drug from the original formulation (sandimmon) (44) and the poor correlation between trough concentration and clinical effects had lead to the reduction of the utility of CsA in therapeutic drug monitoring. During the time, majority of these problems addressed (33); the selected matrix for measurement is blood, not plasma or serum (34,46), the assay for the parent drug are now more selective (47,48) most laboratories participate in proficiency testing (49) and for better absorpsion, the drug has been re-formulated (Neoral1) (50-52). However, trough whole blood concentration remains an imperfect assessment of the total exposure to CsA during a dose interval (53) and a predose blood sample for CsA analysis is used in the was majority of centers.

Lately, it has been approved that capillary blood, gotten by skin puncture is suitable for monitoring CsA (54). This new method can be used specially in pediatric practice.

The relationship between area under the concentration-time curve (AUC) for CsA and clinical events has been characterized by kahan and coworkers in the Sandimmun era (55).They concluded that there is just a poor correlation between trough concentrations and the AUC and thereby do not sufficiently reflect CsA exposure, where as total exposure evaluation (i.e., AUC) would be able to improve correlation with clinical effects of the drug (56,58).

Despite the acknowledgment of AUC monitoring advantages by many scientists, this method has failed to gain worldwide acceptance because of some difficulties, both for the patient and the clinician (59). The measurement of AUC has been simplified by the arrival of the microemulsion formulation of CsA, Neoral, which has the improved pharmacokinetic characteristics, better absorption and bioavailability (59,60). Previous studies with Sandimmun have shown that an accurate estimate of CsA AUC can be got by the concentrations of three blood samples, drawn at specific times (61). For Neoral the same level of accuracy can be obtained by two blood samples, collected within the usual 12 hr dose interval, however, it is conflicting (39,62,63). Interpatient variability still existed and careful TDM and dose adjustment should be performed (64). The comparison of predose concentration monitoring with little or limited sampling AUC monitoring is now waiting for future studies.

However, it should be noticed that the options for the therapeutic monitoring of CsA are not limited only to predose, trough concentration and AUC monitoring.For some time cantarovich and coworkers supported the method of using a single timed sample 6h after dosing (65). In a prospective study for controlling CsA therapy in heart transplant patients, comparison between predose and 6h CsA concentration have been done, the use of 6h value showed a 30%. Lower dose of the drug with the same effectiveness in preventing rejection and the same cardiac and renal function as that seen in those dosed using the predose concentration (66).

These authors have also reported a good correlation between 6h CsA concentrations and efficacy in noninfectious uveitis (67). The usage of CsA blood concentration at 2h postdose is another option for monitoring promotion (C_2_). The reason for this option comes from the observation which was obtained during the clinical development of Neoral when they considered that in liver transplant patients, there was an opposite relationship between the incidence of rejection and the maximum blood CsA concentration (Cmax) (68-70). In a small open-labelled experiment in liver transplant recipients, the usage of C_2_ monitoring showed positive results (71,72). So, the use of C_2_ monitoring has been supported by recent studies of the pharmacokinetics and pharmacodynamics of CsA. In a study on nephritic syndrome patients by Naito and his colleagues, it has been shown that C_1_ and C_2_ are good clinical markers for CsA exposure but not CO (73,74). Pharmacodynamic studies have shown the correlation between the CsA Concentration 2 hr postdose with maximal calcineurin inhibition (75) and maximal reduction in the number of circulating IL-2^+^ CD4^+^ peripheral cells (76). Evidence is also beginning to prove that individualizing a patient’s absorption phase for CsA which called “absorption profiling” by aiming C_2_ concentration results in clinical advantages (77). Different experiments of absorption profiling being conducted in renal, liver and heart transplant patients showed positive results (78,79). But C_2_ can’t be used in all populations-A research in Egypt showed that because of the occurrence of schistosomal infection, Egyptians have special characteristics with regard to drug absorption and metabolism, So C_2_ can be replaced by C_2_5 to monitor CsA (80). Pharmacokinetic studies have shown that the first 4 hr postdose, is the most highly variable region of the blood concentration profile CsA absorption phase between patients (81).

There is also an interaction between cyclosporine and mycophenolate mofetil (MMF), resulted in rise of its concentration and so, reduction of cyclosporine dose may be necessary (82). The correct measurement of cyclosporine has been the subject of many publication (40, 41,83) and reviews (50,84).

As general rule, it is identified that without the advantages of prospective concentration-controlled studies done with validated analytical methodology for CsA in multiple centers, the risk/advantage ratio for specific concentrations of the drug in specific patient group is missing. Eight different immunoassay assay systems for the measurement of CsA in whole blood are now produced by five commercial companies. Furthermore, some laboratories are using high performance liquid chromatography (HPLC) to measure the drug. H. P. L.C has been considered as gold standard in CsA measurement because it is especially possible to couple with mass-spectrometry. The disadvantages of HPLC related to poor precision and fault results because of the interference from other sources (85), of the eight immunoassay variants, two are nonspecific and cross-react, markedly, with the metabolites of cyclosporine. The abbott TD×1 drug and metabolite assay uses a polyclonas antibody and produces results that are approximately 3±5 times that of HPLC. where as the DiaSorin CYCLO-Trac NS radio immunoassay uses a monoclonal nonspecific antibody assays to HPLC changes with the metabolite: parent compound ratio in the blood and therefore will vary with transplant type and time after transplant. The results of the non-specific assays have a poor correlation with clinical events (86). The other six immuno assays are concerned as specific for the parent drug but, to a limited amount, cross react with drugs’ metabolites and therefore do not give the same results for a given sample. It is noticeable that the differences between the results of the specific assay can partly because of the different cross reactivity of antibodies used. The incorrect calibration may lead to some of the differences (87). It is interesting to mention that for one of the manufactures the results of their three different assays do not match. 

It seems that these differences in measurement correctness do not affect the clinical usefulness of the assays (86), but this lead to increase variability of reported concentrations data in the literature (88) and have an impact on the local target ranges. However, in clinical conditions with high load of CsA metabolite in blood, for example, liver transplant patients immediately post transplant, HPLC is the only method which can precisely measure the parent compound (89).

***Tacrolimus***

The food and drug asministration approved tacrolimus (FK-506: Prograf) for prophylaxis of organ rejection in patient receiving allogenic liver transplants. It is generally used in combination with steroids. Tacrolimus is being evaluated in combination with other immunosuppressive agents especially MMF (39) for patients who receive other solid-organ transplants, similar to CsA, too high drug dosage is accompanied with toxicity and too low with rejection. Other similarity is that the whole blood concentration measurement are also used for the monitoring of tacrolimus therapy (90), primary clinical trials which didn’t include concentration monitoring lead to patients with neuro-and nephrotoxicity (91). The pharmacokinetics of tacrolimus is highly variable (92). The rationale for therapeutic drug monitoring of tacrolimus is similar to CsA, because it shares many of the pharmacokinetic and pharmacodynamic problems with CsA. An early observational study on correlation between concentration and effect of the blood concentration in kidney transplant patients who didn’t experience rejection and those who did (93), however, the other more statistically strict studies on kidney and liver transplant patients, showed significant association of low tacrolimus concentrations with rejection and high concentration with nephrotoxicity (94), Although the plausibility of a limited sampling scheme to predict AUC has been investigated (95), the method of choice is still trough, or predose, whole blood concentration the timed samples (96) and AUC monitoring has also been investigated, but unlike CsA, they haven’t been used in clinical practice yet. This may, partly, because of the high relationship observed between trough concentration and Cmax or AUC (39,98).

In a prospective study, one hundred twenty renal transplant patients were chosen for an open label clinical trial which concluded five transplant centers. The patients were categorized randomly to one of three target predose tacrolimus blood blood concentration ranges: Low, middle, or high. Each participating center used quadruple drug therapy, i.e. induction with antilymphocyte globulin and maintenance immunosuppression with tacrolimus, azathioprine, and prednisone. As the result of the 42-day postsurgery study period, the correlation between increasing blood concentration of tacrolimus and (a) the decreasing rate of rejection and (b) the increasing rate of toxicity were both statistically significant (99,100). In some other studies two sampling time points are chosen as a predictable and precise measure of AUCO-12 in stable renal transplant patients (101).

In another open label multicenter prospective study investigating the PRO-Trak 11 ELISA method for tacrolimus measurement in liver transplant patients, one hundred and eleven adult liver transplant patients were chosen at six centers. One of the important results of this research was statistically significant correlation between increasing trough concentrations of tacrolimus and (a) decreasing risk for rejection, according to the lowest blood concentration during the preceding 0 to 7 days, and (b) increasing risk for nephrotoxicity, according to the highest blood concentration during the preceding 7 days (39).

The same cytochrome P_450_ 3A enzyme family which is responsible for biotransformation of CsA, sirolimus and prednisone in enterocytes and liver, metabolize tacrolimus. Nevertheless, these metabolites do not accumulate in blood in most transplant patients to the amount observed for CsA, and the metabolite bias observed with immunoassay methods widely used for tacrolimus measurement in whole blood does not seem to be as problematic for tacrolimus as for CsA. Still, more study data in different patient population will be needed (39,102). Furthermore, tacrolimus metabolism was inhibited by known CYP3A inhibitors such as ketoconazole, cyclosporine A, and nifedipine.

Recent research results on clinical pharmacokinetic show that in transplant patients with CYP3A5 polymorphism the dosage level of tacrolimus must be adjusted (103). As the concentration of tacrolimus found in the blood of stable renal transplant is low, the measurement of the drug became difficult. In-house ELISA, commercial ELISA (Diasorin) and microparticulate enzyme immunoassay, and HPLC. MS (101) methods have been available. The majority of laboratories monitoring tacrolimus use the commercial microparticulate enzyme immunoassay (MEIA. Abbott laboratories) which measures the drug in the range of 3 to 30μg/L and cross react to just small degree of tacrolimus metabolites(102).

***Mycophenolate mofetil (MMF)***

In 1995, for preventing rejection in renal transplant patients, MMF, the morpholinoethyl ester prodrug from mycophenolic acid (MPA) was approved for clinical use. This drug can be combined with CsA and prednisone and act as a pro-drug for that compound (39, 104-106). When taken orally, because of a rapid conversion to MPA by widely distributed esterases, MMF can’t be measured in plasma at any time after oral administration (39,107). In man, MPA is metabolized to 7-O-MPA glucuronide (MPAG) in liver. This molecule is an inactive metabolite that is present in plasma at approximately 20-100 fold higher concentrations than MPA and excreted renally. It was belived that the MPA glucuronide to be the only metabolite of MPA, However, it is known now that there are at least two other metabolites (108). The role of TDM in MMF therapy hasn’t been established yet.

Previously no studies have shown that its concentration correlates either to toxicity or acute rejection, but some new studies have shown that the will be a relationship between MPA pharmacokinetic parameters and clinical outcome (109). Some authors believe that as inter individual pharmacokinetic variability is low, the use of TDM in the great number of patients would be limited (110). In contrast, other authors using the same data, believe that the inter individual pharmacokinetic variability is high and that TDM may play a function role in controlling MMF therapy (111,112).

The latter view was supported by a study of 30 *de novo* heart transplant patients who received tacrolimus and MMF in which the dose of MMF was adjusted to maintain the MPA trough plasma concentration between 2.5 and 4 μg/l (113). These patients were rejection free at 6 months post-transplant and their MMF dose ranged between 0.5 and 6g/day to achieve trough concentration with in a target range. The other helping point is the fact that although the bioavailability of MPA is reported high 94% in healthy subjects and renal transplant patients on an exact dose of MMF 2g/day (114). The magnitude of the AUC range was not reduced by the correction of the AUC range was not reduced by the correction of the AUC values for patient weight (115).

To relate the AUC and Cmax of plasma to the incidence of rejection or toxicity for example its leucopenia, one can use logistic regression and the highly statistically significant relationship was found (110-113). The results gathered from the logistic regression and date from other trials (110) suggest that low plasma MPA AUC is an important risk factor in developing rejection (112, 115,116). A randomized concentration controlled study of MMF in renal transplant patients results confirmed these data (117). The link between high MPA concentrations and adverse effects has not been recognized. The plasma MPA concentration-time profile for a single dose of oral MMF after an overnight fast shows a rapid increase, then a secondary peak at 6-12 hr. This pattern may be considered as an enterohepatic pathway involving MPAG passage into the gastrointestinal tract via biliary excretion, change to MPA via glucoronidase action in gut flora, and reabsorption of the latter into the general circulation (39). 

A retrospective statistical evaluation of MPA dose-interval AUC data correlation with the incidence of acute rejection was performed in patients of a MMF Japanese renal transplant clinical trial (114,118). The study patients were chategorized randomly to one of several doses of MMF, in addition to receiving CsA doses leaded by blood concentration monitoring and empiric doses of prednisone. A significant correlation (*P*= 0.001) was obtained between risk for rejection (relative to the risk with no MMF) and the natural log of the dose-interval MPA AUC, but not to MMF dose (114).

A prospective multicenter randomized concentration-controlled clinical trial in renal transplant patients, sponsored by Roche global development. The patients (n= 5150) from a total of seven citers in and the were categorized randomly to low, intermediate and high target MPA AUC values. A strategy was developed and agreed to permit continual adjustment of dosing to retain the target AUC values with in the 6-month period study: acute rejection incidence and other results were considered. This prospective concentration-clinical response study confirmed the hypothesis of strong statistically significant relationship (*P*= 0.001) between rejection risk and MPA AUC but not MMF dose (118). In another prospective study, they investigated, the use of a 2 hr abbreviated MPA AUC us predose MPA plasma concentrations to control the intrapatient variance of MPA AUC. The relationship between abbreviated MPA AUC and the full 12 hr AUC was very well, but is much more practical to perform in the clinical setting. Another study which was performed on 21 liver transplant recipients children, showed that AUC 0-7 correlated significantly with MMF dose. As MPA pharmacokinetics varies in pediatric liver transplant recipient, monitoring of MPA plasma level is required (119).

Recent studies have determined that there may be a correlation between drug concentration and its toxicity, for example in a study on kidney transplants patients at a fixed dose of 2g/day, a high C (30 min) is accompanied with increased risk of side effects, supporting the idea that dividing the MMF daily oral dose into more than two divided dose might prevent early MPA toxicity (120). Sometimes, MMF administration may be accompanied with tolerability problems. These problems relates to gastrointestinal adverse effects such as nausea, vomiting, diarrhea, abdominal pain, and gastritis. An enteric-coated formulation of mycophenolate sodium (EC-MPS) has been improved to overcome these disorders. EC-MPS releases MPA in the small intestine instead of the enhanced tolerability relative to MMF. In a study performed by cattaneo and his colleagues on stable kidney transplant recipients, the pharmacokinetics of MPA released from new EC-MPS is completely variable and unpredictable, comparing with that after MMF dosing. Despite that there are no significant differences in mean MPA exposure. Expressed as dosage-adjusted MPA AUC 0 to 12 and maximum concentration of drug (Cmax) aberrant kinetic curves in individual patients were found, with an extremely high variability in MPA CO. AUC 0 to 12 and tmax. Moreover, most patients who were given EC-MPS had multiple peaks of MPA in their pharmacokinetic profile that was not seen after long-term MMF administration. These findings were at variance with those of Arns *et al*. showing similar pharmacokinetic profiles after single EC-MPS of MMF administration to kidney transplant patients. Anyway based on these findings, sodium should be taken into account (121), in patients with diabetes.

Some reports suggested that MPA may act with other drugs as well as immunosuppresants (122). The rate of MPA absorption after oral administration of MMF is delayed MPA/MPA glucuronide and tacrolimus may change the rate and amount of MPA absorption because of its prokinetic properties particularly in patients with diabetic gastroparesis. Jeong *et al*. reported that comparing the tacrolimus-based regimen plus standard dose of MMF with CsA-based regimen in renal transplant recipient with diabetes mellitus, showed that MPA exposure was higher in tacrolimus-based regimen. However, changing CsA to tacrolimus didn’t seem to have significant impact on the rate of absorption of MPA, as judged by MPA-Tmax (123,124).

MMF is commonly administered concomitantly with ganciclovir for managing transplant recipients infected with CMV. A study was conducted by Mohammadpour *et al* to evaluate the probable effects of ganciclovir on MPA was not affected by ganciclovir, but ganciclovir increased MPAG AUC and induced entrohepatic recirculation of MPA (125).

Compared with the other immunosuppressant drugs which are currently used, the plasma concentration of MPA is much higher and this makes HPLC measurement of the drug perfom easily. By using this technique the major matbolite MPAG can be resolved and quantified (126).

For accurate and precise measurement of the drug concentration range 0.5-15 mg/ml, use of a commercial homogeneous enzyme immunoassay (Dade Behring) is recommended. (127,128). Because of the cross-reaction of the above antibody, normally, concentrations of MPA measured by HPLC. (129). A new MPA assay based on the enzymatic activity of recombinant IMPDH II (the pharmacological target of MPA) with superb relation with HPLC has been recently produced for the measurement of MPA plasma levels. A study conducted by Marquet *et al* compare this new assay with LC-MS/MS for MPA pharmacokinetic studies in different populations. 

MMF was administrated in association with cyclosporine, tacrolimus or sirolimus. The result showed that foundings were obviously higher that those obtained with LC-MS/MS in patients on cyclosporine or sirolimus, but not in patients on tacrolimus (130).

***Sirolimus***

This drug has been recently approved in USA for use with cyclosporine after kidney transplantation, but it can be used in other clinical indication and with tacrolimus (131). The drug was also approved in where the license specifies its use in the prophylaxis of graft rejection in adult kidney transplant recipients, primarily in combination with CsA and with blood concentration monitoring. 

Therapeutic drug monitoring of sirolimus is still in its primary stage, but data gathered from several clinical trials which were concentration controlled and used sirolimus as primary immunosuppressive therapy (132-134). The measurement of the drug is possible by using HPLC with either mass spectrometric or ultraviolet detection. For pivotal phase III studies an investigational immunoassay was used (135). This immunoassay is no longer available. As a result, attention is now focusing on HPLC techniques for routine monitoring of the drug (136,137). The predose concentrations are generally targeted in the range 4-12 μg/l when sirolimus is used with CsA or tacrolimus.

## Conclusion

We are entering an era in which combination therapy will become routine and clinicians will adjust the immunosuppression to the characteristics of the individual patient, changing dose and drugs as time progresses and conditions change. In conclusion, the knowledge of the pharmacokinetic principles of immunosuppressants is critical for the success after transplantation. The use of TDM as an important treatment strategy for improved outcomes, however, the knowledge about the limitations of TDM is equally important for a continued rational development of immunosuppressive drug therapy after transplantation.
